# Vehicle Trajectory Estimation Based on Fusion of Visual Motion Features and Deep Learning

**DOI:** 10.3390/s21237969

**Published:** 2021-11-29

**Authors:** Lianen Qu, Matthew N. Dailey

**Affiliations:** 1 Department of Information and Communication Technologies, Asian Institute of Technology, Klong Luang, Pathum Thani 12120, Thailand; mdailey@ait.ac.th; 2 International College, Qingdao University of Science and Technology, Qingdao 266061, China

**Keywords:** optical flow, trajectory estimation, Extended Kalman Filter, deep learning, situation awareness, Intelligent Driver Assistance Systems

## Abstract

Driver situation awareness is critical for safety. In this paper, we propose a fast, accurate method for obtaining real-time situation awareness using a single type of sensor: monocular cameras. The system tracks the host vehicle’s trajectory using sparse optical flow and tracks vehicles in the surrounding environment using convolutional neural networks. Optical flow is used to measure the linear and angular velocity of the host vehicle. The convolutional neural networks are used to measure target vehicles’ positions relative to the host vehicle using image-based detections. Finally, the system fuses host and target vehicle trajectories in the world coordinate system using the velocity of the host vehicle and the target vehicles’ relative positions with the aid of an Extended Kalman Filter (EKF). We implement and test our model quantitatively in simulation and qualitatively on real-world test video. The results show that the algorithm is superior to state-of-the-art sequential state estimation methods such as visual SLAM in performing accurate global localization and trajectory estimation for host and target vehicles.

## 1. Introduction

Intelligent Driver Assistance Systems (IDASs) must predict and update a model of the scene around the vehicle in order to anticipate possible future collisions. This requires localization and trajectory estimation, in which we must turn sensor observations into a record of a moving vehicle’s position with respect to the surroundings.

In this paper, we propose a collection of new methods that together enable accurate localization and trajectory estimation for a host vehicle equipped with monocular cameras and target vehicles that are visible around the host vehicle. Localization refers to the sequential state prediction techniques used to turn sensor observations into a record of a moving vehicle’s position with respect to the surroundings. Most approaches to the localization problem combine odometry information with observations of landmarks; examples include SLAM, visual tracking, and 3D reconstruction (structure from motion). The Extended Kalman Filter, particle filter, and grid-based methods are popular state estimation methods for localization. Our primary interest is to combine visual odometry techniques with image-based object detection and target vehicle trajectory estimation, all in a common reference frame, in order to give a driver or an autonomous vehicle accurate situation awareness.

Until recently, image-based object detection was insufficiently accurate and too resource intensive for use in a practical IDAS. However, more recently, the field has greatly improved accuracy and runtime performance, primarily due to better feature extraction, large-scale machine learning models, and low cost high performance embedded Graphical Processing Units (GPU). Early methods for object detection using feature engineering and classical machine learning techniques [1,2,3] have been supplanted by Deep Neural Networks (DNNs) [4,5,6]. With fast, accurate methods such as YOLO [7] now available, it is finally possible to include sophisticated object detectors in an IDAS. However, although we can adopt YOLO for its vehicle detection capabilities, an object detector cannot alone precisely localize a detected vehicle in 3D. We therefore require new algorithms for precise 3D localization of vehicles detected in 2D by YOLO or comparable 2D object detectors.

**Figure 1:**
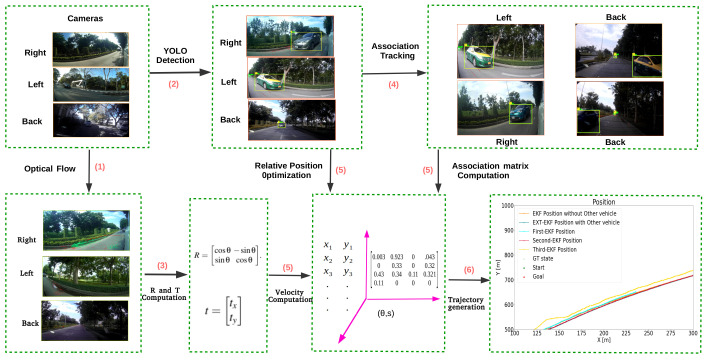
Framework of proposed model. Our experiments use three cameras, one oriented backwards and two oriented approximately $45^{{}^{\circ}}$ outward from the side-view mirrors. The framework estimates the linear velocity and angular velocity of the host vehicle using optical flow (1) followed by RANSAC (3). In parallel, it predicts the relative positions between the host vehicle and any visible target vehicles (2). The method also solves the problem of MTT under conditions in which target vehicles switch from one camera view to another (4). The last step of the method performs sensor fusion using an extended Kalman filter utilizing novel system and sensor models (5). The resulting trajectories are fused into a common world coordinate system (6), then visualized for situation awareness. Note: the data in part (6) are from Experiment II.

To accomplish these goals, we propose a new method for situation awareness in an IDAS that tracks the host vehicle’s velocity and the relative positions of target vehicles around the host vehicle. The overall framework is shown schematically in Figure 1. The algorithm performs these localization and target vehicle trajectory estimation tasks in a common reference frame using the noisy but simple and low cost monocular camera as the only sensor. The contributions are as follows:

1. We introduce a new method for estimation of the host vehicle’s linear and angular velocity that uses optical flow and RANSAC.
2. We introduce a new method for estimating the instantaneous positions of any visible vehicles (target vehicles) relative to the host vehicle using YOLO, a camera calibration model, and a nonlinear optimization procedure.
3. We introduce a new solution to the multiple target tracking (MTT) problem under conditions in which target vehicles switch from one camera view to another.
4. We introduce a sensor fusion method utilizing an extended Kalman filter with novel system and sensor models.

## 2. Related Work

For localization of a host vehicle under mobility in IDASs, Schubert et al. [8] compare different motion models and find that a method assuming a constant yaw rate and acceleration gives the best results. Berthelot et al. [9] and Tamke et al. [10] implement a model for vehicle trajectory estimation. They find that estimated trajectories are precise if the vehicle has movement consistent with the motion estimation model. Huang et al. [11] explore methodologies for vehicle trajectory estimation that rely on differential global positioning systems. Liu et al. [12] describe a trajectory prediction approach that relies on driving behavior prediction and classification using hidden Markov models. Sorstedt et al. [13] consider driver control input parameters to obtain better estimates.

For localization of the host vehicle as well as surrounding vehicles in IDASs, Schreier et al. [14] describe an integrated approach that predicts trajectories based on a maneuver estimation model. Driving maneuvers are inferred for each vehicle with a Bayesian network. Ammoun et al. [15] explore a collision risk estimation model that predicts trajectories of surrounding vehicles.

As input to an IDAS, various sensors could be considered. Morris et al. [16] use LIDAR, which is probably the most useful and reliable sensor for intelligent vehicles, in their vehicle tracking and trajectory estimation system. Dickmann et al. [17] and Clarke et al. [18] use radar in their autonomous driving system and driver assistance systems to model the environment around the vehicle.

For target tracking, those methods can be categorized as either Single Target Tracking (STT) or Multiple Target Tracking (MTT) methods. The simplest and probably the most frequently considered tracking problem is STT. MTT requires locating target positions, maintaining target identities, and generating target trajectories given an input video. Basit et al. [19] employ CAMSHIFT for tracking of a single target for trajectory estimation. Wojke et al. [20] propose deepSORT, an approach rely on the tracking-by-detection paradigm. DeepSORT, when combined with accurate detectors like YOLO, has recently achieved MTT performance that is sufficient for an IDAS.

Additionally, several studies in recent years have analyzed potential applications of trajectory analysis and optimization to improve traffic flow in different scenarios. One example is prediction and planning of Connected Automated Vehicle (CAV) arrivals at intersections followed by optimization of those CAVs’ trajectories through the intersection by an intersection controller [21,22].

Several authors have considered localization of a host vehicle under mobility in IDASs [8,9,10,11,12,13]. These methods require integration of information from multiple sensors. We are interested in how to bypass this limitation by using just a single sensor, the monocular camera, to track the host vehicle’s velocity, and the relative positions of each target vehicle surrounding the host vehicle based on camera data alone.

Various sensors are useful for an IDAS, such as LIDAR [16] and radar [17,18]. Cameras provide better information about object identity than LIDAR or radar, but position accuracy is a challenge.

Some researchers have explored traffic forecasting issues. The goal of traffic forecasting is to predict traffic conditions (e.g., traffic speed, flow, and type of traffic) network-wide based on feeds from real-time traffic sensors, considering spatial and temporal correlations to provide accurate real-time predictions that could be used to guide automated vehicles in real time or city planners over longer terms  [23,24]. The work described in this paper, besides informing drivers of the current real-time situation, could also inform centralized real-time traffic forecasting systems.

Recent monocular Visual Odometry (VO) tracking methods [25,26,27,28,29,30] use deep learning models with a monocular front-facing camera to estimate odometery from visual motion. The limitation of these deep learning methods in performing the VO task is that their knowledge of VO is embedded implicitly in the deep learning model, which as a black box, lacks the explainability of the explicit knowledge represented by the mathematical relationship between optical flow and vehicle motion. Critically, none of this work attempts to track target vehicles along with ego motion, the main focus of our work.

Once objects around the host vehicle are detected, they must be tracked. IDASs require Multiple Target Tracking (MTT). Early methods used classical computer vision techniques [19,31]. DeepSORT has recently achieved MTT performance that is sufficient for an IDAS. We shall see that DeepSORT’s resource utilization is currently prohibitively high for embedded systems usable in an IDAS, and it is unable to process multiple cameras or handle targets that switch between cameras in real time. As our focus is on vehicle trajectory estimation for vehicles traveling on city streets alongside multiple other vehicles using MTT, we propose a new algorithm for tracking the 3D positions of vehicles based on multiple streams of 2D detections that also handles camera switching.

We now describe the proposed algorithm for estimation of host and target vehicle trajectories then present experimental results and conclude the paper.

## 3. Proposed Method

Our method includes four steps: (1) camera calibration, (2) video processing with optical flow to obtain estimates of linear and angular velocity for the host vehicle, (3) target vehicle detection, relative position estimation, and tracking, and (4) fused vehicle trajectory estimation.

### 3.1. Camera Calibration

Points ${\left(X,Y,Z\right)}^{T}$ in camera coordinates are mapped to points ${\left(f_{x}X/Z+u_{x},f_{y}Y/Z+v_{y},1\right)}^{T}$ on the image plane according to a linear mapping in homogeneous coordinates

(1) $$\begin{array}{c} {\left(f_{x}X+Zu_{x},f_{y}Y+Zv_{y},1\right)}^{T}=K\left[I|0\right]X_{cam}. \end{array}$$

We estimate camera parameters $f_{x}$, $f_{y}$, $v_{x}$, and $v_{y}$ along with radial distortion parameters $k_{1}$, $k_{2}$, $k_{3}$ and tangential distortion parameters $p_{1}$ and $p_{2}$ through a standard procedure with a checkerboard. The radial distortion is relatively strong in our lenses, while the tangential distortion is less prevalent. Adding the rotation R and translation of the center of the camera C by −RC, we obtain

(2) x=KR[I|−C]X=K[R|t]X=PX,

where X is an arbitrary 3D point in the vehicle coordinate frame. In order to obtain R and t, we perform an on-vehicle calibration. Using eight manually identified points on the ground and eight corresponding points in the image, R and t are estimated relative to the vehicle frame via Levenberg Marquardt nonlinear least squares minimization of the projection error as implemented by OpenCV’s SolvePnP routine. This gives us a projection matrix P for each camera.

### 3.2. Linear and Angular Velocity Computation from Optical Flow

We use the Lucas-Kanade optical flow algorithm, which is efficient but generates false matches (outliers). This means mismatched points should be removed prior to motion estimation. We use a variant of the random sample consensus (RANSAC) algorithm [32] to remove outliers.

**Figure 2:**
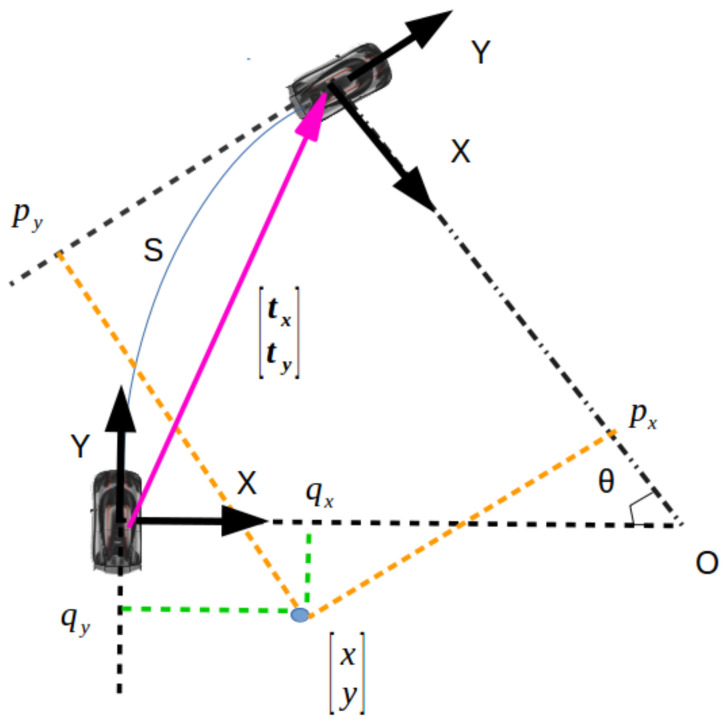
Schematic of vehicle motion model. *s* is the distance traveled (arc length), θ is the angle turned, ${\left[p_{x},p_{y}\right]}^{T}$ is the representation of a world point ${\left[x,y\right]}^{T}$ at time t+1, and ${\left[q_{x},q_{y}\right]}^{T}$ is the representation of ${\left[x,y\right]}^{T}$ at time *t*. ${\left[t_{x},t_{y}\right]}^{T}$ is the origin of the vehicle coordinate system at time t+1 represented in the vehicle coordinate system at time *t*.

Algorithm 1 repeatedly computes optical flow, linear velocity $\dot{s}$, and angular velocity $\dot{\theta }$, accounting for outliers and noisy flows. The method can precisely calculate camera motion without a scale ambiguity, as accurate 3D positions of ground points are known when P is known. RANSAC attempts to find the largest consensus set. If a majority of the points are on the ground, the largest consensus set will necessarily include those points, as their motion will be consistent with just one correct homography that can be calculated from any inlier sample. Neither points off the ground nor incorrect correspondences will have motion consistent with ground plane motion, so they will not be included in the consensus set. To ensure that a majority of optical flows are indeed on the ground, we use ROIs that include mostly ground points in the majority of driving situations see Figure 7 for the RoIs). We estimate host vehicle velocities using optical flows with outliers assumed to be for ground points, so the ROIs should include mostly ground points in the majority of driving situations. On each iteration, we sample two corresponding pairs then compute R and t using the SVD method that follows. In order to compute the rotational and linear velocity of the host vehicle from optical flow, two sets of corresponding inlier points, namely $P_{G}$ and $Q_{G}$, are acquired by steps (b)−(e), from frame $I_{i}$ and $I_{i+1}$, separately. Let $P_{G}=\left\{p_{1},p_{2},...,p_{n}\right\}$ and $Q_{G}=\left\{q_{1},q_{2},...,q_{n}\right\}$ be two sets of corresponding points on the plane. The motion model of the host vehicle is shown in Figure 2. In order to acquire rotation matrix $R_{2\times 2}$ and translation vector $t_{2\times 1}$ that optimally align the two sets $P_{G}$ and $Q_{G}$, corresponding points $p_{i}$ and $q_{i}$ (i∈1,2,…n) should be approximately related by rigid planar motion, i.e.,

(3) $${\left[\begin{array}{c} p_{x}\\ p_{y} \end{array}\right]}_{i}\approx R{\left[\begin{array}{c} q_{x}\\ q_{y} \end{array}\right]}_{i}+t,\;i=1,2,\ldots n.$$

The optimization problem can be considered as

(4) $$R^{*}=\underset{R}{\mathrm{argmin}}||Y-{RX||}_{F},$$

where X is the set $Q_{G}$ arranged as a 2×n matrix and Y is the set $P_{G}$ arranged also as a 2×n matrix. We solve this optimization problem using the SVD, $U\Sigma V^{T}$ = ${\mathrm{XY}}^{T}$, $R=VU^{T}.$
**Algorithm 1:** Velocity from Optical Flow.**Require:** Video $V_{1}$, $V_{2}$, and $V_{3}$ from right, left, and back cameras with regions of interest $R_{1}$, $R_{2}$, and $R_{3}$.**Ensure:**$\dot{\theta }$ (angular velocity), $\dot{s}$ (linear velocity). Procedure:   1.Let t=1 and acquire the first frame $I_{1}^{i}$ of $V_{i}$ in gray scale, i=1,2,3;   2.For each subsequent frame $I_{t+1}^{i}$ of input video, i=1,2,3:  (a)Acquire frame $I_{t+1}^{i}$ in gray scale.  (b)Detect sub-pixel accurate corners in $R_{i}$ for frame $I_{t}^{i}$ to obtain feature set $Q_{I}^{i}$.  (c)Calculate optical flow for $I_{t}^{i}$, $Q_{I}^{i}$, and $I_{t+1}^{i}$ to obtain corresponding sparse feature set $P_{I}^{i}$ using Lucas-Kanade.  (d)Remove keypoints without correspondences from $P_{I}^{i}$ and $Q_{I}^{i}$.  (e)Project $P_{I}^{i}$ and $Q_{I}^{i}$ from the image plane to the ground plane to obtain $P_{G}^{i}$ and $Q_{G}^{i}$ using the camera calibration information (P matrix) determined according to the method described in Section 3.1.  (f)Combine point set $P_{G}^{i}$ and $Q_{G}^{i}$, i=1,2,3, separately to obtain ground point sets $P_{G}$ and $Q_{G}$.  (g)Remove outliers from $P_{G}$ and $Q_{G}$ using RANSAC to obtain consistent ground plane point sets $P_{G}^{\prime }$ and $Q_{G}^{\prime }$.  (h)Compute rotation matrix R and translation vector t using $P_{G}^{\prime }$ and $Q_{G}^{\prime }$.  (i)Compute the rotational velocity $\dot{\theta }$ and linear velocity $\dot{s}$ using R and t. **return** $\dot{\theta }$ and $\dot{s}$.

After acquiring R and t in step (h), the linear and angular velocity of the vehicle over the interval can be calculated simply as

(5) $$\begin{array}{c} \dot{\theta }=\frac{\tan^{-1}\frac{r_{12}}{r_{11}}}{\Delta_{t}},\; \end{array}$$

(6) $$\begin{array}{c} \dot{s}=\frac{r\theta }{\Delta_{t}}, \end{array}$$

where $r_{11}$ and $r_{12}$ are the first two elements of the first row of R. $r=\frac{t_{y}}{\sin\theta }$ is the turning radius. Note that in addition to arc motions, for the particular case of straight motion of the vehicle, θ=0, and the linear velocity can be computed more simply as $\dot{s}=\frac{t_{y}}{\Delta t}$, where $t_{y}$ is the second element of t.

### 3.3. Object Detection and Relative Position Estimation Based on Deep Learning

The goal here is to achieve accurate target vehicle detection and relative position estimation by fusion of CNN-based detection, camera calibration, and optimization. A complete trajectory can be obtained from the dynamic sequence of target vehicle observations over time.

#### 3.3.1. Target Vehicle Detection

In order to perform target vehicle detection in real time, we use the YOLOv3 CNN detection model for detection and classification. YOLOv3 [33] is based on the Darknet 53 CNN and has 106 layers.

#### 3.3.2. 3D Backprojection to Obtain Relative Position

We define two planes, the image plane and the ground plane, and compute a projective transformation from one plane to the other plane. The homography from the image plane to the ground plane is obtained from the projective camera matrix P by removing the third column of P. The process implicitly takes into account the camera’s height, orientation, and position relative to the vehicle frame, as the ground points are specified in the vehicle frame in the on-vehicle calibration process. The relationship can be written

(7) $$\left[\begin{array}{c} u\\ v\\ 1 \end{array}\right]=\lambda H_{GI}X_{G},$$

where λ is an arbitrary scale factor. We also utilize the reverse mapping

(8) $$\begin{array}{c} X_{G}=\frac{1}{\lambda }{\left(H_{GI}\right)}^{-1}\left[\begin{array}{c} u\\ v\\ 1 \end{array}\right]. \end{array}$$

To obtain the relative position of a target, we first guess the relative position of the target based on Equation (8) with the backprojection of the bottom center ${\left[u,v,1\right]}^{T}$ of the bounding box detected by YOLOv3. As the actual position of the target vehicle relative to the backprojection of the bottom center of the bounding box is variable depending on position and orientation, we use the backprojection as an initial guess, but then we refine the guess via optimization. We use Levenberg Marquardt (LM) nonlinear least squares for this purpose. Suppose that the width, length, and height of the target are *w*, *l*, and *h*, respectively. Let the relative position of the target (x,y) be the center of the target cuboid on the ground plane. The eight corners of the cuboid are:

$$\begin{array}{c} \begin{array}{c} \left(x+\frac{w}{2},y+\frac{l}{2},0\right),\left(x-\frac{w}{2},y+\frac{l}{2},0\right),\left(x+\frac{w}{2},y-\frac{l}{2},0\right),\left(x-\frac{w}{2},y-\frac{l}{2},0\right),\\ \left(x+\frac{w}{2},y+\frac{l}{2},h\right),\left(x-\frac{w}{2},y+\frac{l}{2},h\right),\left(x+\frac{w}{2},y-\frac{l}{2},h\right),\left(x-\frac{w}{2},y-\frac{l}{2},h\right). \end{array} \end{array}$$

We project these eight points in 3D to the 2D image. We then pick the four corners of the minimal bounding box enclosing the eight points. The objective function to minimize is the difference between the predicted bounding and the detected bounding boxes in image coordinates, more precisely,

(9) $$\left(x^{*},y^{*}\right)=\underset{\left(x,y\right)}{\mathrm{argmin}}||f\left(x,y\right)-z{\left|\right|}^{2},$$

where z contains the *u*, *v* coordinates of the four corners of the bounding box detected by YOLOv3. f(x,y) reprojects the 3D points to 2D points in the image and calculates the minimum enclosing bounding box.

### 3.4. Visual Tracking and Camera Switch Processing

Target detection and target observation were discussed in Section 3.3. In this section, we describe how the tracker maintains vehicle identity and handles camera switches for targets.

#### 3.4.1. Visual Track Handling

The tracking method integrates the predicted state ${\hat{x}}_{t}$, the detected bounding box $a_{i}$ for each detection *i*, and the predicted bounding box $b_{j}$ for each tracked target *j*. In the system state, estimates of the host vehicle and target vehicles’ positions and velocities are recorded. In order to determine whether to match YOLO detection $a_{j}$ with predicted bounding box $b_{j}$ calculated from the system state for target $a_{j}$, we compute the IoU (Intersection over Union) cost for the two bounding boxes:

(10) $$S_{IoU}=1-\frac{A\left(a_{i}\right)\cap A\left(b_{j}\right)}{A\left(a_{i}\right)\cup A\left(b_{j}\right)},$$

where A(a) gives the set of pixels included in bounding box *a*. We repeat the calculation for all $a_{i},i\in 1\ldots n$ and $b_{j},j\in 1\ldots m$. We use the Hungarian algorithm to find the association minimizing the total cost. For predicted targets that are matched, we save the new detection’s bounding box with the correct track data structure. For unmatched boxes, we create new track data structures and associated Kalman filter state variables. The Kalman filter will on subsequent observations update the target vehicle states using the predicted bounding boxes. Tracks for vehicles unmatched for $t_{d}$ frames are deleted from the Kalman filter’s state vector and covariance matrix. We found empirically that when a vehicle is missed for seven or more frames, it is rarely recovered by the tracker. We therefore set $t_{d}=7.$

#### 3.4.2. Camera Switches

**Figure 3:**
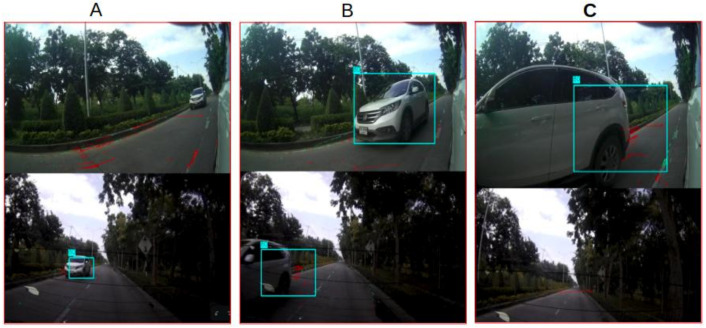
Example target tracking and camera switching. (**A**) Example image set $I_{t}$ containing one target in backward camera. (**B**) Example image set $I_{t^{\prime }},t^{\prime }>t$ containing the same target in the backward and right cameras. The area of the bounding box in right camera is larger than that in the backward camera. At that point, tracking switches from the backward to the right camera. (**C**) The target has disappeared in the backward camera but is tracked in the right camera.

To ensure consistent detection of all nearby vehicles, the system should be designed with overlapping fields of view between cameras. As target vehicles in overlapping fields of view may be detected in multiple cameras, we ensure that the tracker only uses one of the three cameras, based on a heuristic evaluation of which camera should be used to track. The heuristic is the size of the detected bounding box in each camera. Each target vehicle is tracked in the camera for which its predicted bounding box is largest in the image. An example is shown in Figure 3.

### 3.5. Vehicle Trajectory Estimation

We employ an extended Kalman filter to fuse the host vehicle’s linear and angular velocity with measured relative positions of target vehicles.

#### 3.5.1. Vehicle State

The vehicle state $x_{t}$ describes the host vehicle’s instantaneous position, linear velocity, and angular velocity, as well as target vehicles’ positions and velocities in the world coordinate system:

$$\begin{array}{c} x_{t}={\left[\begin{array}{c} x_{t}^{h},y_{t}^{h},\theta_{t}^{h},{\dot{\theta }}_{t}^{h},{\dot{s}}_{t}^{h},x_{t}^{1},y_{t}^{1},{\dot{x}}_{t}^{1},{\dot{y}}_{t}^{1},\ldots ,x_{t}^{n},y_{t}^{n},{\dot{x}}_{t}^{n},{\dot{y}}_{t}^{n} \end{array}\right]}^{T}, \end{array}$$

where $\left(x_{t}^{h},y_{t}^{h}\right)$ is the host vehicle’s position, $\theta_{t}^{h}$ is the host vehicle’s rotation in the world coordinate system, ${\dot{\theta }}_{t}^{h}$ is the host vehicle’s 2D rotational velocity, and ${\dot{s}}_{t}^{h}$ is the host vehicle’s linear velocity. $\left(x_{t}^{i},y_{t}^{i}\right)$ and $\left({\dot{x}}_{t}^{i},{\dot{y}}_{t}^{i}\right)$ are the position and velocity of vehicle *i*. *n* is the number of vehicles. Since the number of vehicles being tracked varies dynamically over time, the state vector and associated covariance matrices are expanded or collapsed as needed when new vehicle tracks are created or destroyed. The system model is assumed to be

(11) $$\begin{array}{c} x_{t+1}=f\left(x_{t}\right)+v_{t}, \end{array}$$

where $x_{t}$ and $x_{t+1}$ are the state at times *t* and t+1, and $v_{t}∼N$$\left(0,Q_{t}\right)$ is a Gaussian random vector modeling the randomness in the state transition. f(·) is the system transition function.

To define f(·), ${\dot{\theta }}_{t}$ is the rotational velocity around the *z* axis in the host vehicle coordinate system. The orientation $\theta_{t+1}$ in the world coordinate system at time t+1 must therefore be

(12) $$\begin{array}{cc} \; & \theta_{t+1}=\theta_{t}+{\dot{\theta }}_{t}\Delta_{t}. \end{array}$$

The actual steering angular and linear velocity are not measured, so we assume any change in linear or rotational velocity to be noise. The host vehicle velocity parameters can thus be expressed as

(13) $$\begin{array}{c} {\dot{\theta }}_{t+1}={\dot{\theta }}_{t} \end{array}$$

(14) $$\begin{array}{c} {\dot{s}}_{t+1}={\dot{s}}_{t}. \end{array}$$

With finite *r*, the vehicle’s displacement in the vehicle coordinate system is described by

(15) $$\begin{array}{c} \left[\begin{array}{c} t_{x}\\ t_{y} \end{array}\right]=r\left[\begin{array}{c} 1-\cos\dot{\theta }\Delta_{t}\\ \sin\dot{\theta }\Delta_{t} \end{array}\right]. \end{array}$$

For the particular case of straight movement, we can obtain

(16) $$\begin{array}{c} \left[\begin{array}{c} t_{x}\\ t_{y} \end{array}\right]=\left[\begin{array}{c} 0\\ \dot{s}\Delta_{t} \end{array}\right]. \end{array}$$

The host vehicle’s position at time t+1 can be described as

(17) $$\begin{array}{c} \left[\begin{array}{c} x_{t+1}\\ y_{t+1} \end{array}\right]=\left[\begin{array}{c} x_{t}\\ y_{t} \end{array}\right]+\left[\begin{array}{cc} \cos\theta_{t} & -\sin\theta_{t}\\ \sin\theta_{t} & \cos\theta_{t} \end{array}\right]\left[\begin{array}{c} t_{x}\\ t_{y} \end{array}\right]. \end{array}$$

For the other vehicles, we assume linear dynamics

(18) $$\begin{array}{c} \left[\begin{array}{c} x_{t+1}^{i}\\ y_{t+1}^{i}\\ {\dot{x}}_{t+1}^{i}\\ {\dot{y}}_{t+1}^{i} \end{array}\right]=\left[\begin{array}{c} x_{t}^{i}\\ y_{t}^{i}\\ {\dot{x}}_{t}^{i}\\ {\dot{y}}_{t}^{i} \end{array}\right]+\left[\begin{array}{c} \Delta_{t}{\dot{x}}_{t}^{i}\\ \Delta_{t}{\dot{y}}_{t}^{i}\\ 0\\ 0 \end{array}\right]. \end{array}$$

To approximate nonlinear function f(·), we linearize around an arbitrary point ${\hat{x}}_{t}$, i.e.,

(19) $$\begin{array}{c} f\left(x_{t}\right)\approx f\left({\hat{x}}_{t}\right)+J_{f}\left({\hat{x}}_{t}\right)\left(x_{t}-{\hat{x}}_{t}\right). \end{array}$$

Here $J_{f}\left({\hat{x}}_{t}\right)$ is the Jacobian evaluated at ${\hat{x}}_{t}$.

(20) $$J_{f}\left({\hat{x}}_{t}\right)=\left[\begin{array}{c} \frac{\partial f}{\partial x_{t}}{|}_{\hat{x}} \end{array}\right]$$

#### 3.5.2. Observation Model

We incorporate optical flow tracking of points on the ground and the deep learning model capable of producing a prediction of the position of neighboring vehicles’ projections into the image plane at time *t* to obtain estimated observations

$$\begin{array}{c} z_{t}=\left[\begin{array}{c} {\dot{\theta }}_{t}^{\prime },{\dot{s}}_{t}^{\prime },x_{t}^{\prime 1},y_{t}^{\prime 1},\ldots ,x_{t}^{\prime n},y_{t}^{\prime n} \end{array}\right],\;i=0,1,\ldots n, \end{array}$$

where ${\dot{\theta }}_{t}^{\prime },$ and ${\dot{s}}_{t}^{\prime }$ are the measured angular and linear velocity of the host vehicle, respectively. $\left[x_{t}^{\prime i},y_{t}^{\prime i}\right]$ are the measured relative offsets between the host vehicle and vehicle *i*. We define the observation with an equation h(·) mapping the vehicle state $x_{t}$ to the corresponding observation $z_{t}$:

(21) $$\begin{array}{c} z_{t}=h\left(x_{t}\right)+w_{t}, \end{array}$$

where $w_{t}∼N$$\left(0,S_{t}\right)$.

The homogeneous representation of target *i*’s bottom center ${\left[x_{t}^{v},y_{t}^{v},1\right]}^{T}$ in the vehicle coordinate system, assuming a flat ground plane, is calculated as

(22) $$\begin{array}{c} \left[\begin{array}{c} x_{t}^{vi}\\ y_{t}^{vi}\\ 1 \end{array}\right]=T_{t}^{w/v}\left[\begin{array}{c} x_{t}^{i}\\ y_{t}^{i}\\ 1 \end{array}\right]. \end{array}$$

Here the rigid transformation $T_{t}^{w/v}$ maps from the world coordinate system to the vehicle coordinate system at time *t*. The calculation can be repeated for each vehicle i∈1,…,n. In detail, if we obtain a rotation matrix $R_{t}$ from the vehicle’s orientation $\theta_{t}^{h}$ at time *t*, the transformation matrix $T_{t}^{w/v}$ is

(23) $$\begin{array}{c} T_{t}^{w/v}=\left[\begin{array}{cc} R_{t} & -R_{t}\left[\begin{array}{c} x_{t}^{h}\\ y_{t}^{h} \end{array}\right]\\ 0 & 1 \end{array}\right]. \end{array}$$

Similar to the transition model f(·), we linearize $h(x_{t})$ around arbitrary point ${\hat{x}}_{t}$ using the Jacobian evaluated at ${\hat{x}}_{t}$:

(24) $$J_{h}\left({\hat{x}}_{t}\right)=\left[\begin{array}{c} \frac{\partial h}{\partial x}{|}_{{\hat{x}}_{t}} \end{array}\right].$$

#### 3.5.3. Initialization

To initialize the system state, we assume the host vehicle’s initial trajectory is accurately represented by the optical flow between two initial frames. We calculate the angular velocity and linear velocity as discussed in Section 3.2. We construct the first observation $z_{0}$ without including other vehicles in the system state:

$$\begin{array}{c} z_{0}=\left[\begin{array}{c} {\hat{\theta }}_{0},{\hat{s}}_{0} \end{array}\right]. \end{array}$$

The initial state is obtained from $z_{0}$, considering the position of the host vehicle to be the origin at time t=0. The initial state vector is thus

$$\begin{array}{c} x_{0}={\left[0,0,0,{\hat{\theta }}_{0},{\hat{s}}_{0}\right]}^{T}. \end{array}$$

Whenever new vehicles are detected, the observation $z_{t}$ includes the optimized position of the vehicle relative to the host vehicle. The world position and orientation of the host vehicle are initialized to zero, while the velocities of the host vehicle are initialized from $z_{t}$ at time *t*, written as

$$\begin{array}{c} z_{t}=\left[\begin{array}{c} {\hat{\theta }}_{t},{\hat{s}}_{t},{\hat{x}}_{t}^{1},{\hat{y}}_{t}^{1},\ldots ,{\hat{x}}_{t}^{n},{\hat{y}}_{t}^{n} \end{array}\right],\;n=0,1,\ldots n. \end{array}$$

We likewise augment the state estimate $x_{t}$ with new entries for the new vehicle’s position and velocity. We initialize the velocity of each target vehicle using the first two frames in which it is detected.

#### 3.5.4. Noise Parameters

For the system and sensor noise, the Kalman filter imposes the assumption of Gaussian noise. We parameterize these noise distributions assuming reasonable correlations between the system state and the noise. We assume that the observation noise is a linear function of linear velocity, turning angle rate, and the other vehicles’ relative positions in the vehicle coordinate system. We assume $R_{t}$ is diagonal. We let the entries of $R_{t}$ corresponding to the angular and linear velocity be ${\left(\alpha_{1}+\alpha_{2}{\hat{\dot{\theta }}}_{t}\right)}^{2}$ and ${\left(\alpha_{3}+\alpha_{4}{\hat{\dot{s}}}_{t}\right)}^{2}$. We let the entries of $R_{t}$ corresponding to the relative position be ${\left(\alpha_{5}+\alpha_{6}{\hat{x}}_{t}^{i}\right)}^{2}$ and ${\left(\alpha_{7}+\alpha_{8}{\hat{y}}_{t}^{i}\right)}^{2}$.

We also suppose for simplicity that $Q_{t}$, the state transition noise covariance, is diagonal. For the host vehicle, the $Q_{t}$ elements corresponding to the positions are $\Delta_{t}^{2}\left(\eta_{1}\hat{\dot{s}}+\eta_{2}\right)$ and $\Delta_{t}^{2}\left(\eta_{1}\hat{\dot{s}}+\eta_{2}\right)$. The $Q_{t}$ element corresponding to the rotation is $\Delta_{t}^{2}\left(\eta_{3}\hat{\dot{\theta }}+\eta_{4}\right)$. The $Q_{t}$ element corresponding to the angular velocity is $\Delta_{t}^{2}\left(\eta_{5}\hat{\dot{\theta }}+\eta_{6}\right)$. The $Q_{t}$ element corresponding to the linear velocity is $\Delta_{t}^{2}\left(\eta_{7}{\dot{s}}_{t}^{h}+\eta_{8}\right)$. For the other vehicles, the $Q_{t}$ elements corresponding to the position are $\Delta_{t}^{2}\left(\eta_{9}{\dot{x}}_{t}+\eta_{10}\right)$ and $\Delta_{t}^{2}\left(\eta_{11}{\dot{y}}_{t}+\eta_{12}\right)$. The $Q_{t}$ elements corresponding to the velocity of target vehicles are $\Delta_{t}^{2}\left(\eta_{13}{\dot{x}}_{t}+\eta_{14}\right)$ and $\Delta_{t}^{2}\left(\eta_{13}{\dot{y}}_{t}+\eta_{14}\right)$. These noise distributions are simplistic and ignore many factors, but they suffice for the experiments in this paper. The parameters’ values are determined through a combination of analytical and empirical methods. We begin with reasonable values based on analysis of the underlying noise source, then we validate the parameters first in simulation and then in the real world. The parameters used in the experiments are as follows: $\alpha_{1}=\alpha_{2}=0.01$, $\alpha_{3}=\alpha_{4}=0.02$, $\alpha_{5}=\alpha_{7}=0.6$, $\alpha_{6}=\alpha_{8}=1$, $\eta_{1}^{2}=\eta_{2}^{2}=0.2$, $\eta_{3}^{2}=\eta_{4}^{2}=0.01$, $\eta_{5}^{2}=\eta_{6}^{2}=0.01$, $\eta_{7}^{2}=\eta_{8}^{2}=0.05$, $\eta_{9}^{2}=\eta_{10}^{2}=0.2$, $\eta_{11}^{2}=\eta_{12}^{2}=0.2$ and $\eta_{13}^{2}=\eta_{14}^{2}=1$. These specific values reflect some tuning to obtain smooth trajectories in the simulation experiments, but no changes were necessary to obtain the reported results for the real-world experiments.

$P_{0}$ denotes the initial state error. We propagate the observation error for $z_{0}$ through $h(x_{0})$ and take into account the initial uncertainty about the optical flow.

#### 3.5.5. Update Algorithm

The system state model and observation model are described in Section 3.5, Equations (11) and (21). The main difference with an ordinary Kalman filter is that when updating the system state, the prediction method of the Extended Kalman Filter can be propagated without an observation correction to deal with cases in which optical flow fails due to key point mismatches or features undetected in the field of view. Under such conditions, we assume the observation $z_{t}$ is not available, so we estimate the system state and allow propagation (diffusion) of the state covariance without an observation update. This means only the prediction step is implemented, without a correction, when optical flow is unavailable.

## 4. Results and Discussion

We evaluate the proposed model with five experiments. All simulations were implemented in Python with synthetic data. First, we perform a simulation to estimate the robustness of the proposed method for linear and angular velocity estimation for the host vehicle. The simulation allows analysis with different noise levels and different outlier ratios for synthetic optical flow. Our method is compared with a state of the art visual odometry method, ORB-SLAM. Second, we perform a simulation to quantitatively evaluate the accuracy of trajectory estimation for the host vehicle and target vehicles. Third, we evaluate the target vehicle tracker module under real-world motion between cameras. This experiment provides a quantitative assessment of the system’s ability to handle camera switches for target vehicles. Fourth, we perform experiments that combine optical flow, YOLOv3, Hungarian association, and the Extended Kalman Filter for estimating the trajectories of the host vehicle and target vehicles in the real world. Since there is no ground truth, the evaluation is qualitative, but integrated real-world runs do indicate the effectiveness of the method. Finally, we compare estimated trajectories generated by the proposed method with those of ORB-SLAM. This provides evidence of the proposed method’s robustness compared to the predominant alternative method for ego-motion estimation based on monocular cameras. All of the experiments were implemented using C++, the OpenCV library, and Python. We tested the runtime performance of the proposed model on a 2.50 GHz × 4 Intel Core i5 laptop running 64-bit Ubuntu Linux with an NVIDIA GeForce MX150 as well as on an NVIDIA Jetson TX2.

In order to evaluate the proposed method, we prepared two video datasets for Experiment V. We mounted three cameras, one oriented backwards at the top of the rear windshield and two cameras oriented at approximately $45^{{}^{\circ}}$ from the side-view mirrors. The first dataset was collected on a long, nearly straight road in a college campus. We recorded from three cameras concurrently during driving time of approximately five minutes. Thirty six target vehicles appeared in the field of view of one or more cameras during the run. The second dataset was collected on a winding road comprising a straight segment of 100 m, a left turn of 150 m, a straight segment of 150 m, and then a turn right of 100 m. Twenty two target vehicles appeared in the field of view of one or more cameras during this run. Although there were no sudden sharp turns, the winding curves were sharp enough to disrupt monocular visual SLAM but did not disrupt optical flow.

### 4.1. Experiment I (Velocity Estimate Comparison Using Simulation Data)

We conducted a simulation in which we compared the proposed method’s estimates of host vehicle velocity to those of visual SLAM under various noise levels and outlier ratios with synthetic optical flow feature point generation.

#### 4.1.1. Results

The experimental conditions and outcomes we obtained are shown in Table 1. In each simulation, we specify a hard-coded ground truth trajectory, then we use that trajectory to synthesize optical flows for random points on the ground plane. We give ORB-SLAM sufficiently clean initialization data that it can estimate an exact initial homography, thereby addressing monocular SLAM’s inherent scale ambiguity. We add Gaussian noise and outliers to the synthetic data in the synthetic image plane. Since we generally observe real-world outlier ratios between 6% and 69%, in the simulation experiments, we perform experiments with two levels of outlier ratio: 40% and 70%. We add Gaussian random noise to each image point with σ = 1.0, 2.0, or 3.0.

#### 4.1.2. Discussion

The results show that for Gaussian random noise with σ=1.0 and σ=2.0, our proposed method is better than visual SLAM. When the noise levels are more substantial (σ=3.0), visual SLAM is better than the proposed method.

### 4.2. Experiment II (Trajectory Estimation in Simulation)

Besides instantaneous velocity estimation, to verify the correctness and effectiveness of the overall trajectory estimation approach, we evaluated predicted trajectories quantitatively in four simulations.

#### 4.2.1. Results

The first two simulations were performed without target vehicles, and the last two simulations included target vehicles. A detailed list of experimental conditions and outcomes is shown in Table 2.

In each simulation, we generated ground truth and noisy observation data for the host vehicle and target vehicles. For the host vehicle, the data consist of synthetic optical flow for the scene surrounding the host vehicle, from which the method must estimate odometry (angular and linear velocity). For the target vehicles, the data are exact (run 3) or noisy (run 4) observations of target vehicles’ simulated relative positions assuming appropriate velocities. During the simulation, target vehicles are assumed to be correctly detected inside each camera’s field of view until they exit the camera view.

**Figure 4:**
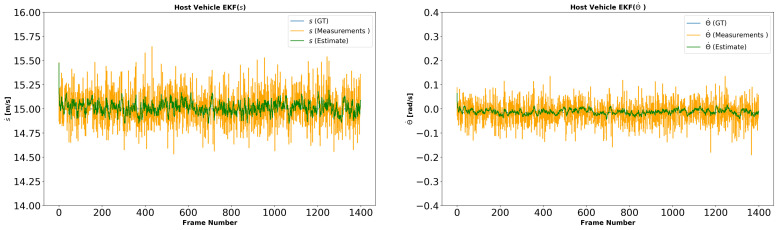
Experiment II (velocity estimation in simulation) results. Left: $\dot{s}$ is the linear velocity of the host vehicle. Right: $\dot{\theta }$ is the angular velocity of the host vehicle. Ground truth is a constant 15 m/s and −0.01 rad/s. Green lines show estimated values from the proposed model. Orange lines are the noisy observations. The filter is effective at smoothing the velocity of the host vehicle.

**Figure 5:**
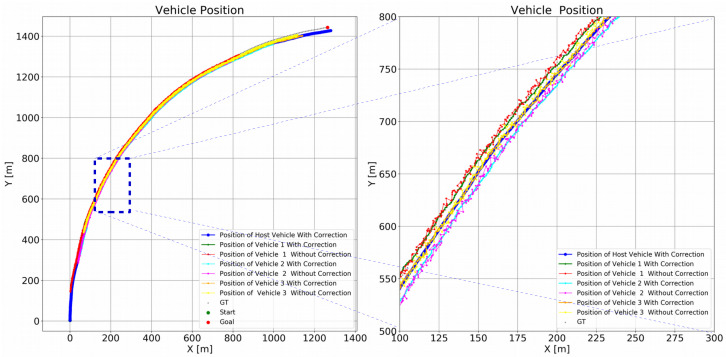
Experiment II (trajectory estimation in simulation) results. Global location of the host vehicle and three target vehicles with different speeds are shown. The host and three target vehicles followed the gray paths. Absolute trajectory estimates are biased because of accumulated host vehicle positioning error. Noise was added to optical flow and the detected relative positions of the target vehicles. The blue path shows the estimated host vehicle path.

**Figure 6:**
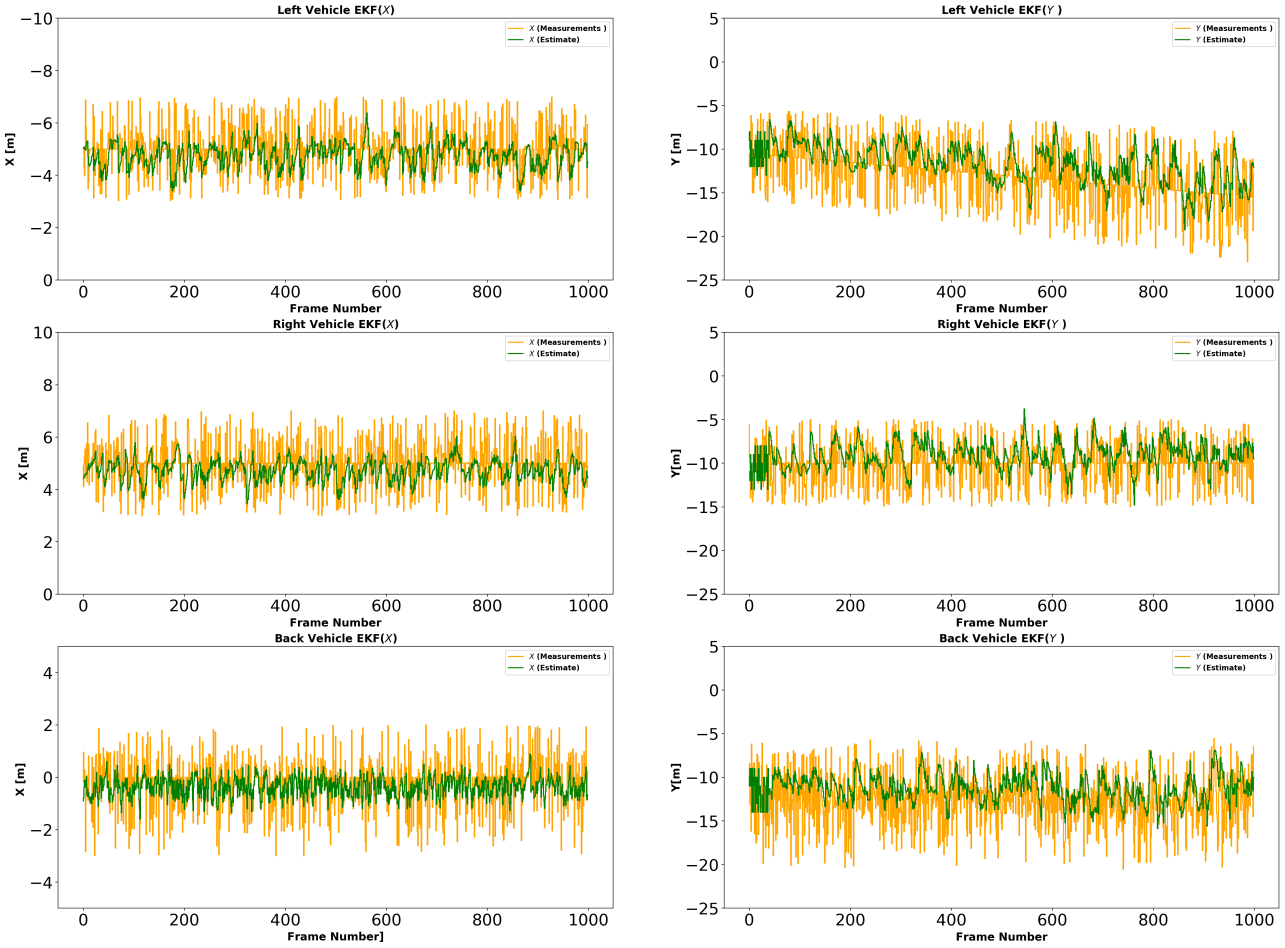
Experiment II (trajectory estimation in simulation) results. Relative position of host vehicle and target vehicles estimated by the proposed model. Relative position error with sensor-only observation is shown in orange, and relative position error after correction by the model is shown in green. We observe that the filter is able to smooth the relative positions of the target vehicles. The green, cyan, and orange paths show target vehicle paths with filtering. The red, magenta, and yellow lines are the estimated paths of the target vehicles without filtering. The graphic on the right magnifies the graphic on the left in the range: x ∈ [100…300], y ∈ [500…800]. Target vehicle paths are smoother under the proposed method.

In each simulation, to model the sensor observations, we first project the synthetic data into the image plane. We use 10% outliers in the optical flow. We add Gaussian random noise to each optical flow point in each image with σ=2.0 and use the noisy data to compute the velocity of the host vehicle. We reproject image points to the ground plane to compute $\dot{\theta }$ and $\dot{s}$ according to the method described in Section 3. In simulation 3 and 4, we add three target vehicles with three different speeds to the simulation. The speed of the target vehicle on the left is slower than that of the host vehicle. The speed of the target vehicle on the right is faster than the host vehicle. The speed of the target vehicle behind the host vehicle matches the host vehicle. The estimated host vehicle velocities in the first two simulations are shown in Figure 4. The estimated host vehicle and target vehicle trajectories over the fourth simulation are shown in Figure 5. The estimated relative positions of the three target vehicles over the fourth simulation are shown in Figure 6.

#### 4.2.2. Discussion

This experiment allows us to compare the performance of sensor-only estimation and model-based correction under the same conditions, with the simulation of optical flows proceeding the same as in Experiment I, except that we also simulate ground truth trajectories and noisy YOLO detections for a set of target vehicles. Clearly, the estimated relative positions are substantially smoother than the raw observations.

### 4.3. Experiment III (Visual Tracking Evaluation)

In this experiment, We tested the target vehicle tracker in a real world environment with vehicles moving between camera views. As a point of comparison, one of the best known state-of-the-art multiple object tracking methods is DeepSORT. To determine the relative accuracy and resource utilization of our method compared to the state of the art, we compare the proposed model to DeepSORT in terms of accuracy and frame rate, on both an unconstrained system and a resource-constrained system.

#### 4.3.1. Results

**Figure 7:**
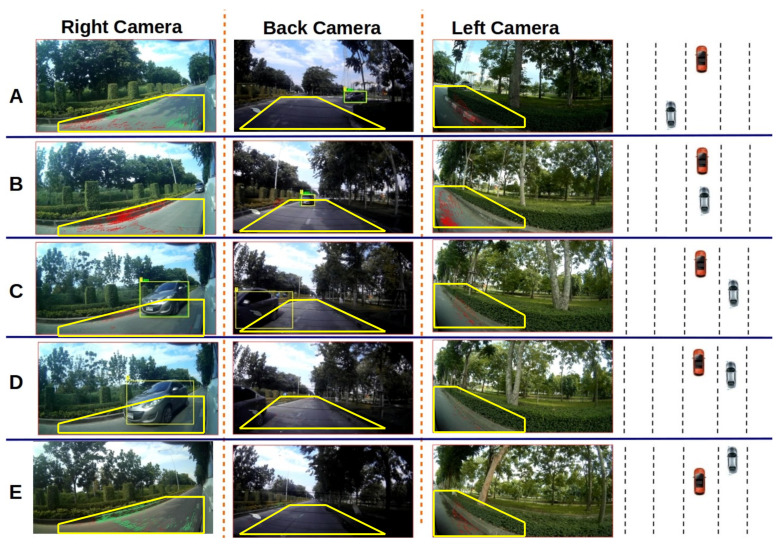
Experiment III (visual tracking evaluation) results. Row (**A**): example images in which a vehicle is detected and tracked by the back-side camera. This target was also visible in the left-side view but was not detected. Row (**B**): example images acquired when the vehicle exits the left-side camera view and enters the right-side and the back-side cameras views. This target vehicle was tracked in the back-side camera. Row (**C**): example images acquired as the vehicle moves from the back-side view to the right-side camera view. This vehicle was detected in frame 259 in the right-side view. Switching to the right-side camera is successful. Row (**D**): example image set acquired after the target has exited the back-side view. This vehicle continues to be tracked by the right-side camera. Row (**E**): example image set in which the target has exited the field of view in the right camera. This vehicle was mis-detected in the backward camera at frames 234, 235, and 236. It was then re-detected and tracked successfully.

Results are shown in Table 3 and Figure 7. Table 3 lists the results of three separate runs on different roads. Figure 7 shows image sets and tracking results for one run. In the run shown in Figure 7 on the first frame, two vehicles are detected in the backward camera, and tracking begins. Target No. 1 appears in the three cameras, one after another.

#### 4.3.2. Discussion

On the unconstrained system, an Intel i5 with GPU, DeepSORT’s tracking accuracy is almost identical to our method’s accuracy, as they are both tracking by detection methods using the same detector (YOLOv3). DeepSORT has a nearly negligible advantage in ID switches, as its deep association metric is sometimes able to maintain track identity when multiple vehicles cause a tracking error in our system. However, the processing speed of the proposed model is substantially faster than DeepSORT on the Intel system. The mean speed of our proposed method is approximately 30 fps, whereas the speed of DeepSORT is about 21 fps. On the NVIDIA Jetson TX2, which we set up to concurrently process streams from three cameras, the processing speed for our method was approximately 5 fps for each of three concurrent video streams (15 fps total). But with DeepSORT as the tracker, we found that the system cannot run alongside the detector and optical flow modules concurrently, due to memory constraints. An additional limitation is that DeepSORT cannot deal with target vehicle switching between cameras. In contrast, the proposed system explicitly deals with parallel processing of three cameras with overlapping views and seamlessly processes target switches between cameras. We conclude that our tracker provides near state-of-the-art accuracy with far less resource utilization than DeepSORT.

### 4.4. Experiment IV (Velocity and Trajectory Estimation in Real World)

In comparisons between estimated target vehicle position and ground truth, we find that the maximum error in relative position estimation is 5.3%. From these results, we conclude that the accuracy of the camera calibration and the relative localization method based on camera calibration is sufficient for our purposes.

#### 4.4.1. Results

**Figure 8:**
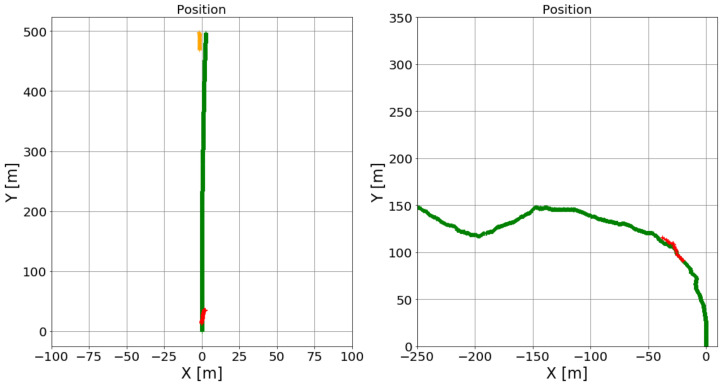
Experiment IV (velocity and trajectory estimation in real world) results. The proposed model was tested in a real-world environment. Host vehicle ran with three target vehicles on two different roads. The left graphic shows a scenario in which the host vehicle was driven on a straight road. The graphic shows the paths of the host vehicle and two example target vehicles. The green line shows the host vehicle path on the road. The red and orange lines show the first and second target vehicle paths, separately. The first target vehicle moves from the left side to the right side of the host vehicle. Later, another target vehicle passes the host vehicle on its left side. The right graphic shows one host vehicle and one target vehicle. The host vehicle was driven on a ‘S’ shaped curved road. The green and red lines show the host vehicle and target vehicle path, separately. The target vehicle follows the host vehicle for some time and then passes the host vehicle on the right.

**Figure 9:**
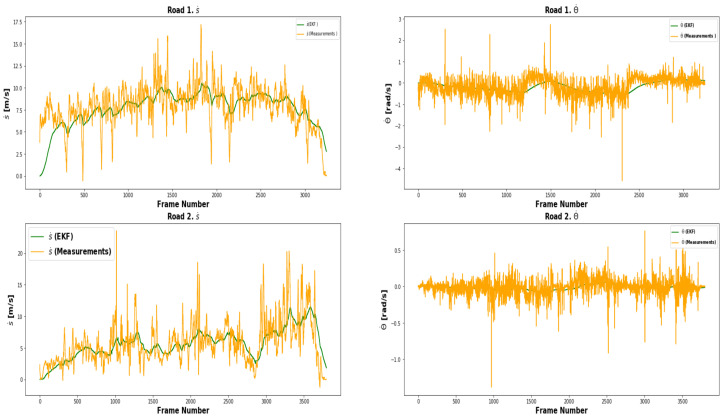
Experiment IV (velocity and trajectory estimation in real world) results. The proposed tracking algorithm was tested in a real-world environment with target vehicles on two roads. Upper left: linear velocity on road 1. Upper right: angular velocity on road 1. Lower left: linear velocity on road 2. Lower right: angular velocity on road 2. The velocity (linear and angular) with sensor-only observations is shown in orange, and relative positions after correction by the model are shown in green. We observe that the filter is able to smooth the linear and angular velocity of the host vehicle.

As it is difficult to obtain ground truth data under real world conditions, we perform a qualitative evaluation of the integrated system’s ability to predict the host vehicle’s velocity and host and target vehicle trajectories in real scenes. In Figure 8 and Figure 9, we show estimated velocities and trajectories for the host vehicle and some example targets in the world coordinate system.

In each run, the host vehicle starts parked. We record this initial position as (0,0), and we compute the velocity of the host vehicle starting from the second frame of optical flow. Target vehicle positions are initialized from the second frame they are detected in. We use YOLOv3 to detect target vehicles, we use the camera calibration model to measure relative position, and we use the Hungarian algorithm to associate detections with tracks.

#### 4.4.2. Discussion

The proposed approach provides less noisy, more smooth, and more stable estimates of the vehicle’s trajectory than the raw observations. During tracking, some targets are missed temporarily. This is due to partial occlusion. In order to deal with this problem, we perform system state prediction without an update computation in the Kalman filter.

### 4.5. Experiment V (Comparison between Proposed Method and Visual-SLAM)

To compare the performance of the proposed approach with a state-of-the-art monocular visual odometry method, we carried out two tests.

#### 4.5.1. Results

**Figure 10:**
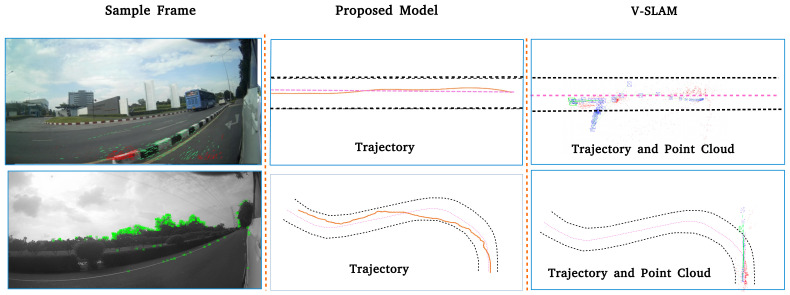
Experiment V (comparison between proposed method and visual SLAM) results. Row 1 shows results for a long straight road. Black dotted lines indicate the border of the road. Pink dotted lines indicate the ground truth center of the road. Orange lines indicate estimated trajectories of the host vehicle. ORB-SLAM was not able to detect features in frames 2790-3245. When ORB-SLAM relocalizes the vehicle, it estimates an incorrect trajectory for the host vehicle. The proposed model, on the other hand, is able to maintain a much more accurate trajectory. Row 2 shows results for a winding road. In this condition, ORB-SLAM works for only a short period of time. After that, it falls and cannot relocalize. The proposed model maintains a smooth trajectory until the host vehicle parks. We conclude that the proposed model for trajectory estimation of the host vehicle is better than ORB-SLAM in outdoor road environments.

We compare the results of our model with a ROS-based monocular visual SLAM method, ORB-SLAM [34]. For the ground truth, we manually traced the host vehicle’s trajectories on satellite maps. We qualitatively compare results from the proposed method to those of ORB-SLAM. ORB-SLAM generates a sparse feature point cloud as the map then localizes the camera within that map for each keyframe. The sequence of keyframe estimates give the estimated vehicle trajectory. The results of the experiment are displayed in Figure 10.

#### 4.5.2. Discussion

They show that our approach is more robust and provides a good approximation to the vehicle’s trajectory in real world driving situations. ORB-SLAM, on the other hand, is only able to generate an estimated trajectory for the host vehicle, not for target vehicles.

## 5. Conclusions

This paper presents a new method for estimating vehicle trajectories from video sequences captured by moving cameras without additional sensors. The host vehicle’s instantaneous velocity is estimated using optical flow with RANSAC. Target vehicles in the frame are detected by a deep learning model. Relative positions of target vehicles are obtained using a perspective transformation and a new optimization method. We use an extended Kalman filter to track host vehicle linear velocity, host vehicle angular velocity, and relative positions of target vehicles, resulting in precise host and target vehicle trajectory estimates in a common world coordinate system. In a series of experiments, we show that the new method substantially reduces the trajectory errors, relative position estimation errors, linear velocity errors, and angular velocity errors inherent in the use of a noisy sensor. The new method also compares favorably against state of the art visual odometry and tracking methods in terms of accuracy and resource utilization. We conclude that the method is an extremely good candidate for the next phase of commercial exploitation, given sufficient compute power.

The main limitation of the method is the assumption that the host vehicle and all target vehicles move on a common flat ground plane. In the current version of the system, when this assumption is violated, such as when the host vehicle goes over a speed bump, the estimated velocity of the host vehicle will be erroneous, and estimated relative positions of the vehicle will vary from the ground truth. In future work, we plan to eliminate this limitation, further test the algorithm on a vehicle-based embedded camera system, experiment with more sophisticated trackers, and develop a reasonably priced consumer grade real time display for driving situation awareness.

## Figures and Tables

**Table 1:** Experiment I (linear and angular velocity accuracy) results. $L\_{\mathrm{RMSE}}_{\mathrm{pg}}$ (m/s) compares predicted values to ground truth for linear velocity using the full system. $L\_{\mathrm{RMSE}}_{\mathrm{mg}}$ (m/s) compares raw observations without Kalman filtering to ground truth for linear velocity. $A\_{\mathrm{RMSE}}_{\mathrm{pg}}$ (rad/s) compares predicted values to ground truth for angular velocity. $A\_{\mathrm{RMSE}}_{\mathrm{mg}}$ (rad/s) compares raw observations to ground truth for angular velocity. Values are means plus or minus standard deviations over 10 runs. The simulation uses a constant host vehicle angular velocity of $\dot{\theta }=-0.01$ rad/s and a linear velocity of $\dot{s}=15$ m/s. OR = Outlier Ratio, GN = Gaussian Noise, P = Proposed Model, O = ORB-SLAM.

OR	GN	$\mathrm{PL}\_{\mathrm{RMSE}}_{\mathrm{pg}}$	$\mathrm{PL}\_{\mathrm{RMSE}}_{\mathrm{mg}}$	$\mathrm{OL}\_{\mathrm{RMSE6}}_{\mathrm{pg}}$	$\mathrm{PA}\_{\mathrm{RMSE}}_{\mathrm{pg}}$	$\mathrm{PA}\_{\mathrm{RMSE}}_{\mathrm{mg}}$	$\mathrm{OA}\_{\mathrm{RMSE}}_{\mathrm{pg}}$
0	σ=0	0	0	0.050±0.003	0	0	0.008±0.003
	σ=1.0	0.070±0.003	0.197±0.008	0.188±0.004	0.020±0.003	0.051±0.002	0.030±0.002
40%	σ=2.0	0.171±0.006	0.466±0.025	0.189±0.002	0.032±0.003	0.120±0.006	0.035±0.002
	σ=3.0	0.285±0.009	1.400±0.064	0.199±0.003	0.088±0.029	0.172±0.005	0.051±0.004
	σ=1.0	0.082±0.005	0.236±0.010	0.176±0.010	0.019±0.002	0.062±0.002	0.049±0.002
70%	σ=2.0	0.204±0.008	0.970±0.010	0.220±0.015	0.094±0.002	0.148±0.002	0.084±0.003
	σ=3.0	0.336±0.021	6.411±0.032	0.231±0.032	0.101±0.003	0.215±0.021	0.091±0.001

**Table 2:** Experiment II (trajectory estimation in simulation) results. ${\mathrm{RMSE}}_{\mathrm{pg}}$ compares predicted values to ground truth, ${\mathrm{RMSE}}_{\mathrm{mg}}$ compares raw observations to ground truth.

Host Vehicle	Target Vehicle(s)	Performance
Noise: 0 Angular velocity: $\dot{\theta }=-0.01$ rad/s. Linear velocity: $\dot{s}=15$ m/s.	No vehicle	Estimated host vehicle path tracks ground truth path perfectly. Angular and linear velocity track the ground truth. Host vehicle position: ${\mathrm{RMSE}}_{\mathrm{pg}}$ = 0, ${\mathrm{RMSE}}_{\mathrm{mg}}$ = 15,613.221.
	No vehicle	Host vehicle’s path is tracked smoothly. Host vehicle position: ${\mathrm{RMSE}}_{\mathrm{pg}}$ = 25.236, ${\mathrm{RMSE}}_{\mathrm{mg}}$ = 21,236.324.
Angular velocity: $\dot{\theta }=-0.01$ rad/s. Linear velocity: $\dot{s}=15$ m/s. Percentage of outliers: 10%. Noise: Gaussian with $Q_{t}$ fixed.	Three vehicles are simulated. Left vehicle: x = −5 m, y = −10 m; Right vehicle: x = 5 m, y = −10 m; Back vehicle: x = 0 m, y = −11 m. Noise: 0.	Host vehicle path is tracked smoothly, and target vehicles’ paths fit the host vehicle’s path. Host vehicle position: ${\mathrm{RMSE}}_{\mathrm{pg}}$ = 64.231, ${\mathrm{RMSE}}_{\mathrm{mg}}$ = 2,121,545.342. Target vehicle relative position: ${\mathrm{RMSE}}_{\mathrm{pg}}$ = 3.341, ${\mathrm{RMSE}}_{\mathrm{mg}}$ = 0.
	Three vehicles are simulated as above with noise: x: 40%, y: 50%.	Details are given in Section 3. Host vehicle position: ${\mathrm{RMSE}}_{\mathrm{pg}}$ = 47.34, ${\mathrm{RMSE}}_{\mathrm{mg}}$ = 24,456.63. Target vehicle relative position: ${\mathrm{RMSE}}_{\mathrm{pg}}$ = 2.272, ${\mathrm{RMSE}}_{\mathrm{mg}}$ = 5.59.

**Table 3:** Experiment III (tracking model) results. TFs = Total Frames, GT IDs = Number of Ground Truth IDs, IDS = Number of ID Switches, P = Proposed Model, DS = DeepSORT, SSBC = Number of Successful Switches Between Cameras.

Place	Camera	TFs	GT IDs	P IDS	P SSBC	DS IDS	DS SSBC
	left		2	0		0	
I	right	3245	17	2	19	2	0
	back		35	6		4	
	left		0	0		0	
II	right	1493	11	3	11	3	0
	back		25	4		2	
	left		0	0		0	
III	right	2236	6	2	6	2	0
	back		16	4		3	

## Data Availability

Not applicable.

## Abbreviations

The following abbreviations are used in this manuscript:

EKF	Extended Kalman Filter
IDASs	Intelligent Driver Assistance System
MTT	Multiple Target Track
OR	Outlier Ratio
GN	Gaussian Noise
P	Proposed Model
O	ORB SLAM
L_RMSE	RMSE of linear velocity
A_RMSE	RMSE of angular velocity
pg	Predicted values to ground truth
mg	Measurement values to ground truth.
TFs	Total Frames
GT IDs	Number of GroundTruth IDs
IDS	Number of ID Switches
DS	DeepSORT
SSBC	Number of Successful Switches Between Cameras
